# Supplementary material for: An Interpretable 3D Bag-Of-Visual-Words Pipeline for Volumetric Microscopy Classification
Source: bioRxiv. 2026 Apr 22:2026.04.21.719969. Preprint. [Version 1] doi: 10.64898/2026.04.21.719969 (PMC13131622; doi:10.64898/2026.04.21.719969)
Supplement: Supplement 1 [file NIHPP2026.04.21.719969v1-supplement-1.pdf]

bioRxiv preprint doi: https://doi.org/10.64898/2026.04.21.719969; this version posted April 22, 2026. The copyright holder for this preprint (which was not certified by peer review) is the author/funder, who has granted bioRxiv a license to display the preprint in perpetuity. It is made available under aCC-BY 4.0 International license.

SI Figures

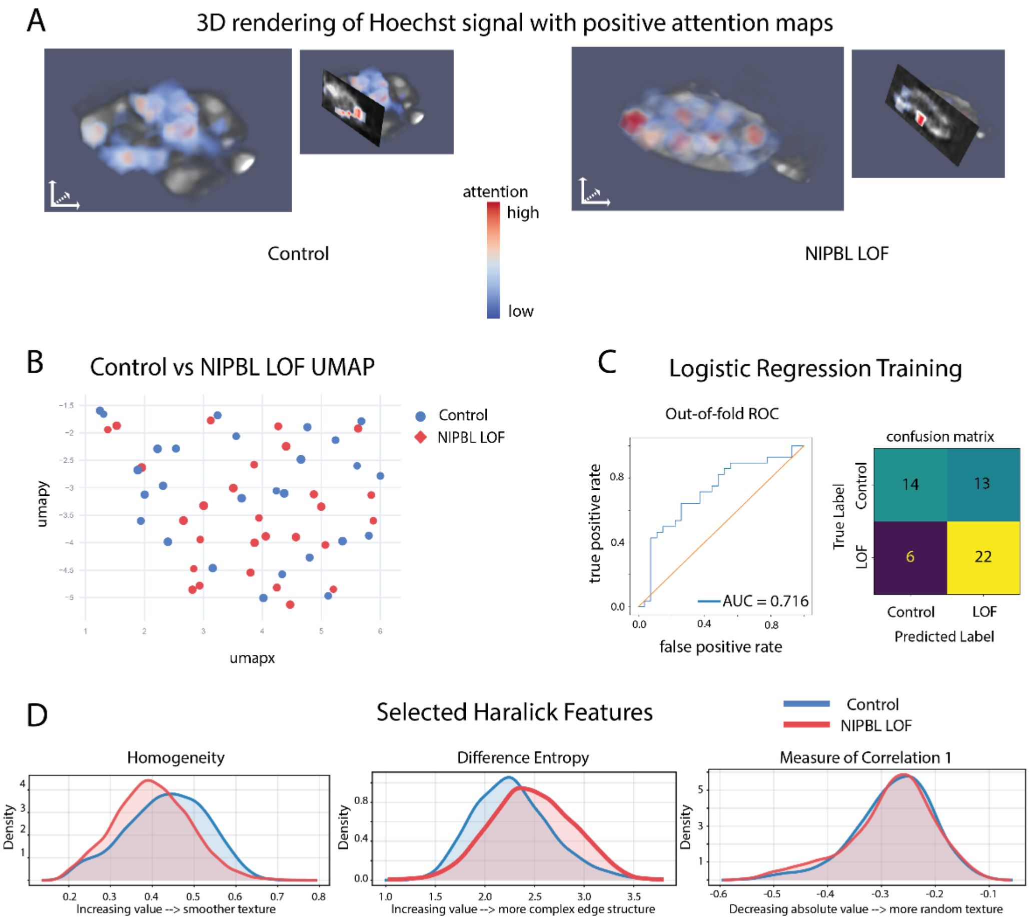

SI Figure 1 ***Hoechst results*** *A: 3D renderings of the Hoechst channel overlaid with the positive attention maps to highlight areas of high attention. B: UMAP results from the normalized image embedding vectors C: A logistic regression model was trained on the image labels and normalized embedding vectors. The model trained with an AUC-ROC of 0.716. D: Selected kernel density estimate graphs from various Haralick Features. The KDEs were generated by analyzing patch-level Haralick feature values and summing over the two conditions.*

bioRxiv preprint doi: https://doi.org/10.64898/2026.04.21.719969; this version posted April 22, 2026. The copyright holder for this preprint (which was not certified by peer review) is the author/funder, who has granted bioRxiv a license to display the preprint in perpetuity. It is made available under aCC-BY 4.0 International license.

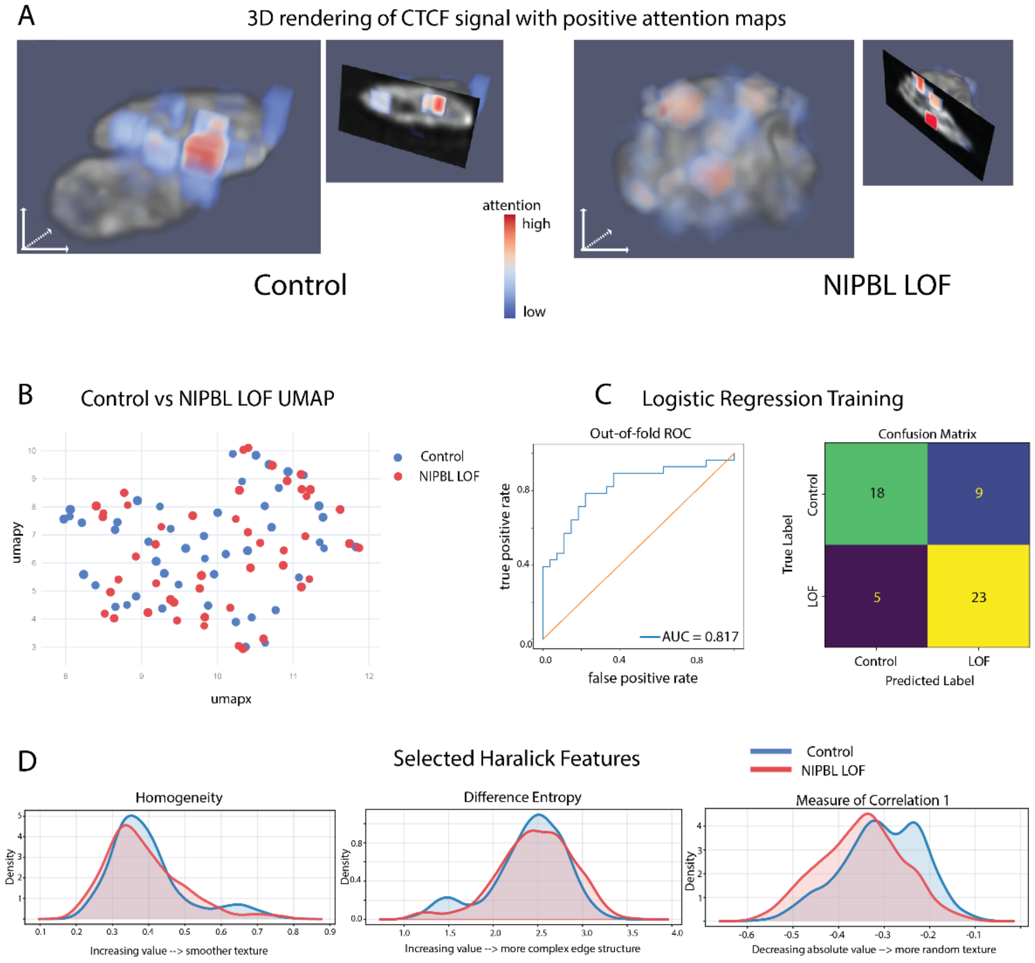

SI Figure 2 ***CTCF Results*** *A: 3D renderings of the CTCF channel overlaid with the positive attention maps to highlight areas of high attention. B: UMAP results from the normalized image embedding vectors C: A logistic regression model was trained on the image labels and normalized embedding vectors. The model trained with an AUC-ROC of 0.817. D: Selected kernel density estimate graphs from various Haralick Features. The KDEs were generated by analyzing patch-level Haralick feature values and summing over the two conditions.*

bioRxiv preprint doi: https://doi.org/10.64898/2026.04.21.719969; this version posted April 22, 2026. The copyright holder for this preprint (which was not certified by peer review) is the author/funder, who has granted bioRxiv a license to display the preprint in perpetuity. It is made available under aCC-BY 4.0 International license.

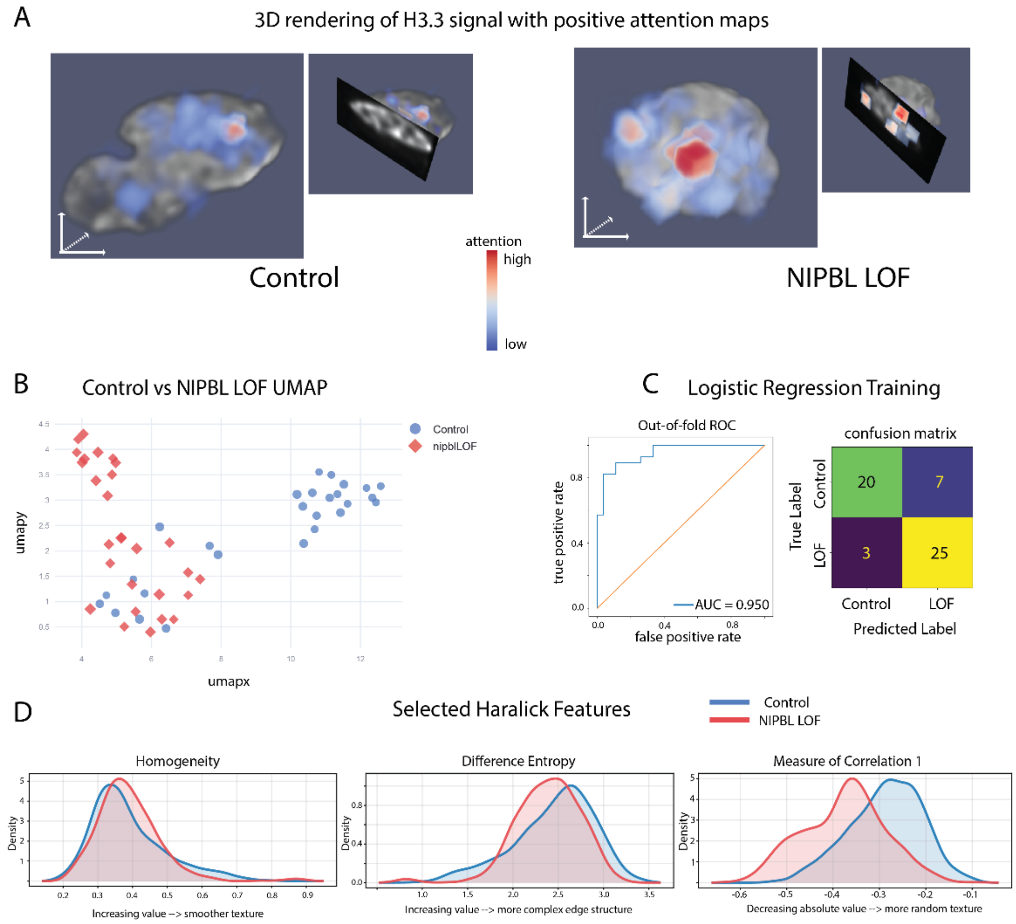

SI Figure 3 ***H3.3 Results*** *A: 3D renderings of the H3.3 channel overlaid with the positive attention maps to highlight areas of high attention. B: UMAP results from the normalized image embedding vectors C: A logistic regression model was trained on the image labels and normalized embedding vectors. The model trained with an AUC-ROC of 0.950. D: Selected kernel density estimate graphs from various Haralick Features. The KDEs were generated by analyzing patch-level Haralick feature values and summing over the two conditions.*

bioRxiv preprint doi: https://doi.org/10.64898/2026.04.21.719969; this version posted April 22, 2026. The copyright holder for this preprint (which was not certified by peer review) is the author/funder, who has granted bioRxiv a license to display the preprint in perpetuity. It is made available under aCC-BY 4.0 International license.

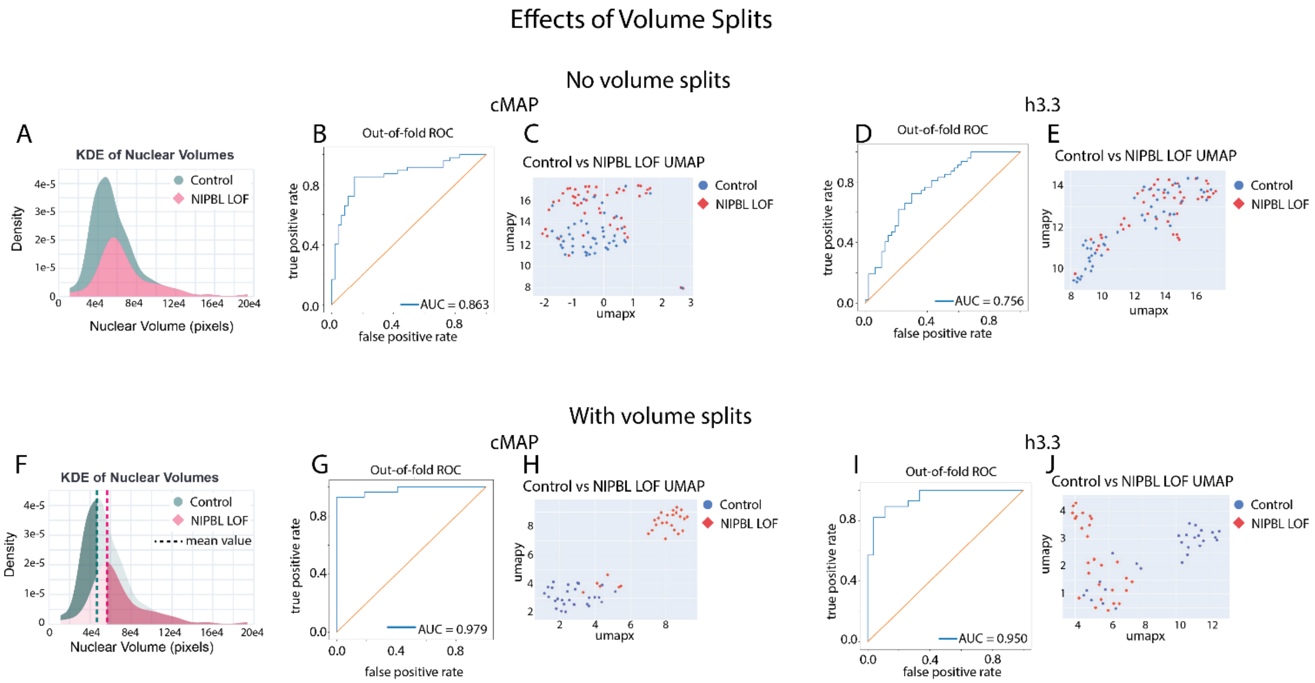

SI Figure 4 ***Effects of volume splits on classification****. Analyses were performed either on the full dataset without volume splitting (top row, A–E) or after size stratification (bottom row, F–J), using smaller control nuclei and larger NIPBL LOF nuclei split at the median nuclear volume, as described for the chromatin dataset analyses. A and F, kernel density estimates of nuclear volume distributions for control and LOF nuclei; F shows the mean volume for each condition and the applied split. B and G, out-of-fold ROC curves for logistic regression models trained on normalized BoVW vectors from the facultative heterochromatin channel, with AUC increasing from 0.863 to 0.979 after stratification. C and H, corresponding UMAP embeddings for facultative heterochromatin. D and I, out-of-fold ROC curves for logistic regression models trained on H3.3 BoVW vectors, with AUC increasing from 0.756 to 0.950 after stratification. E and J, corresponding UMAP embeddings for H3.3. These results show that volume-based stratification reduces overlap between control and NIPBL LOF nuclei and improves condition separation in both channels.*

bioRxiv preprint doi: https://doi.org/10.64898/2026.04.21.719969; this version posted April 22, 2026. The copyright holder for this preprint (which was not certified by peer review) is the author/funder, who has granted bioRxiv a license to display the preprint in perpetuity. It is made available under aCC-BY 4.0 International license.

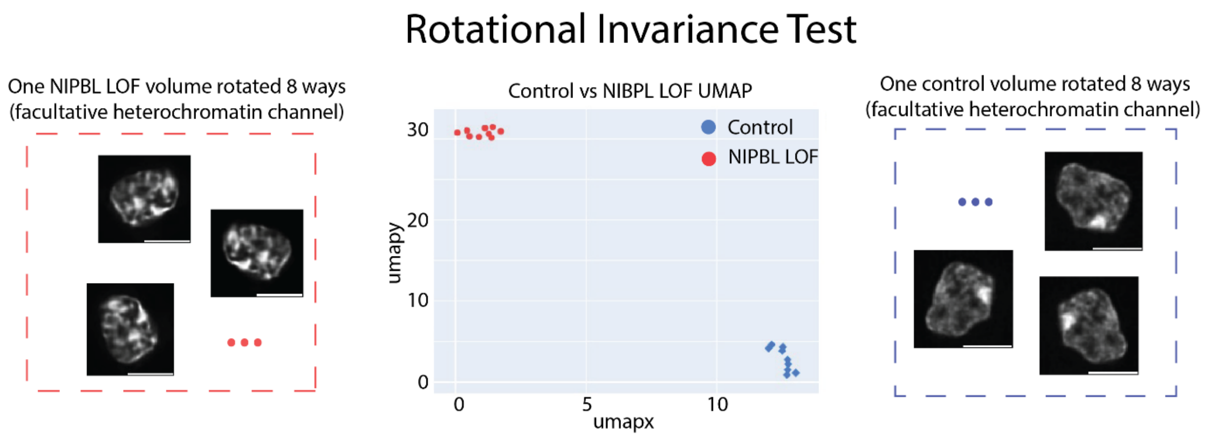

SI Figure 5 ***Rotational Invariance****. A minimal test dataset was generated from two individual facultative heterochromatin biosensor nucleus crops, one control and one NIPBL LOF. For each volume, eight rotated variants were created and analyzed together with the original image using the full pipeline. The resulting UMAP embedding shows that rotated versions cluster with their corresponding source image rather than separating by orientation, consistent with the rotationally robust descriptor design described in the manuscript*

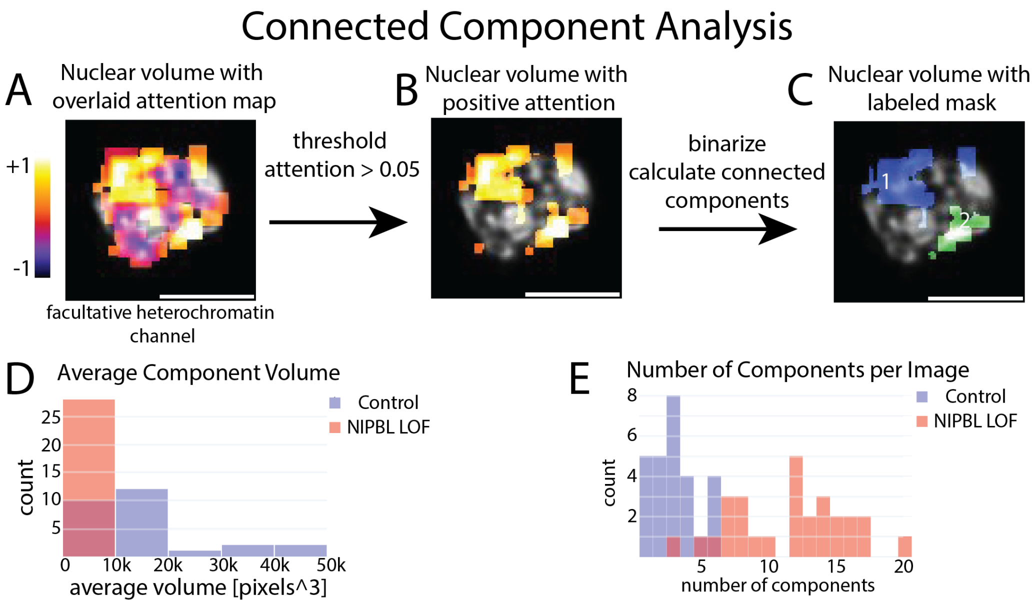

SI Figure 6 ***Connected-component ("blob") analysis of positive attention regions in facultative heterochromatin biosensor expressing CGNs.*** *A, representative nucleus overlaid with the attention map, with positive and negative patch contributions shown on a signed scale. B, positive attention regions retained after thresholding the attention map (> 0.05). C, binary mask after connected-component labeling, with individual high-attention components identified for downstream quantification. D, distribution of average connected-component volume per image for control and NIPBL LOF nuclei. E, distribution of the number of connected components per image for each condition. Consistent with the attention-map trends described in the manuscript, control nuclei tended to contain fewer, larger connected high-attention regions, whereas NIPBL LOF nuclei showed a greater number of smaller, more fragmented high-attention regions*

bioRxiv preprint doi: https://doi.org/10.64898/2026.04.21.719969; this version posted April 22, 2026. The copyright holder for this preprint (which was not certified by peer review) is the author/funder, who has granted bioRxiv a license to display the preprint in perpetuity. It is made available under aCC-BY 4.0 International license.

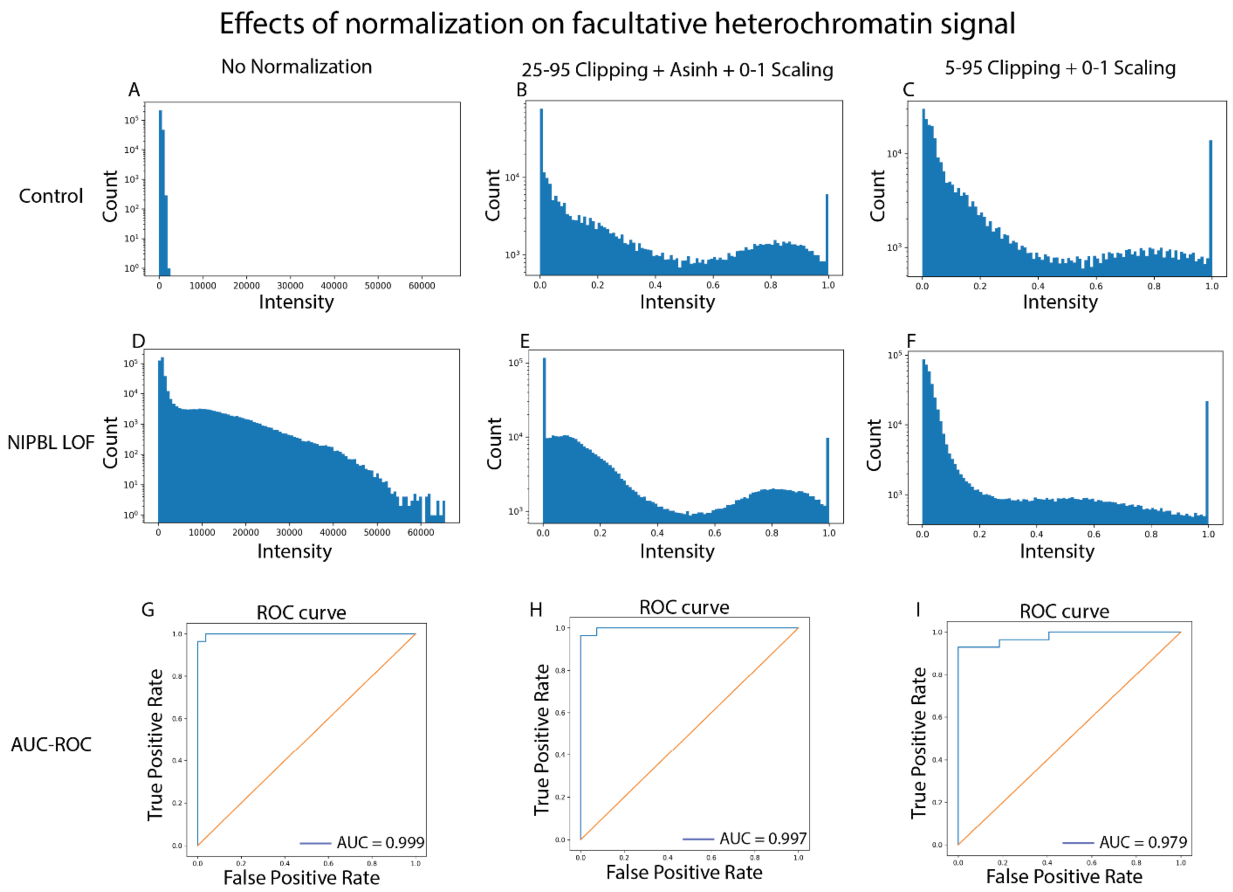

SI Figure 7 ***Effects of normalization on image intensity****. A-C, intensity histograms of the facultative heterochromatin channel from a selected control image under various normalization conditions. D-F, intensity histograms of the facultative heterochromatin channel from a selected NIPBL LOF image under various normalization conditions. G-I, ROC curves from training the LR model on the entire dataset under various normalization conditions.*

bioRxiv preprint doi: https://doi.org/10.64898/2026.04.21.719969; this version posted April 22, 2026. The copyright holder for this preprint (which was not certified by peer review) is the author/funder, who has granted bioRxiv a license to display the preprint in perpetuity. It is made available under aCC-BY 4.0 International license.

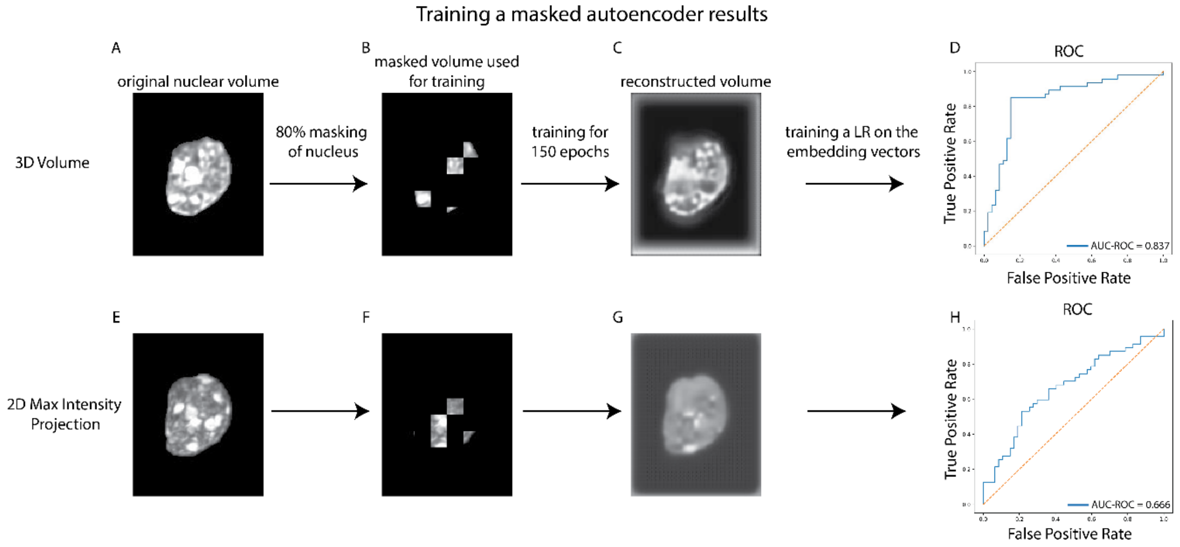

SI Figure 8 ***Comparison with embedding vectors from custom trained masked autoencoder.*** *Full facultative heterochromatin biosensor channel nuclear volumes, or 2D maximum intensity projections, were used to train a custom masked autoencoder (MAE) to generate embedding vectors to compare to the embedding vectors from the Bag-of-visual-words pipeline. The MAE was implemented as a 3D convolutional masked autoencoder (ConvMAE3D; base width 64) trained with 80% blockwise masking (4 × 16 × 16 voxels) for 150 epochs using AdamW (learning rate = 1 × 10* $^{-4}$*) and masked L1 reconstruction loss; image-level embeddings were obtained by mean-pooling bottleneck features for downstream logistic regression. A,C: original images (volumetric or 2D max projection). B,F: images after 80% masking was done. Only the nucleus was masked to avoid having the MAE learn background textures. C,G: reconstruction results after 150 epochs of training. D,H: the embedding vectors from the trained MAE were used to train a 2-class logistic regression model in the same way as with the BoVW vectors in the paper. Both cases (volumetric and 2D) did not perform as well as the BoVW vectors.*
